# A Novel Antimicrobial Peptides From Pine Needles of *Pinus densiflora* Sieb. et Zucc. Against Foodborne Bacteria

**DOI:** 10.3389/fmicb.2021.662462

**Published:** 2021-05-20

**Authors:** Junho Lee, Hee Kyoung Kang, Hyeonsook Cheong, Yoonkyung Park

**Affiliations:** ^1^Department of Biomedical Science, Chosun University, Gwangju, South Korea; ^2^Research Center for Proteineous Materials (RCPM), Chosun University, Gwangju, South Korea

**Keywords:** *Pinus densiflora*, pine needles, antimicrobial peptide, ultrafiltration, foodborne bacteria

## Abstract

Pine needles are used in several East Asian countries as food or traditional medicine. It contains functional components that exhibit a wide spectrum of pharmacological effects such as antioxidant, antimicrobial, anti-diabetic, and anti-inflammatory activities. We determined and characterized the novel antimicrobial peptides (AMPs) isolated from *Pinus densiflora* Sieb. et Zucc. The four active pine-needle (PN) peptides showed antimicrobial activity against foodborne bacteria with minimum inhibitory concentration (MIC) values within the range of 8–128 μg/ml. PN peptides showed no detectable hemolytic activity or cytotoxicity at the antimicrobial concentrations. The *N*-terminal amino acid sequence of the PN5 was identified using Edman degradation and Antimicrobial Peptide Database (APD) homology analysis showed that it was not identical to any other plant peptide. This suggests that PN5 can serve as an alternative therapeutic agent to be used in the food industry.

## Introduction

Antimicrobial peptides (AMPs) play an important element in the innate immune system ranging from bacteria to plants, mammals, and insects (Hancock et al., 2016; Haney et al., 2019; Sathoff and Samac, 2019). Innate immunity is a defensive response in all multicellular organisms to combat pathogens. AMPs involved in these processes typically have a broad-spectrum activity against a wide range of Gram-positive and Gram-negative bacteria, fungi, and even certain viruses (Coates et al., 2018; Mookherjee et al., 2020). In the majority of cases, the mode of action of AMPs is related to the cytoplasmic membrane permeabilization. Plants are a source of bioactive compounds with various properties that are applicable in agriculture and medicine. Plants produced are short AMPs with a molecular mass less than 10 kDa; structurally, these are amphipathic and generally positively charged molecules at physiologically neutral pH values. They primarily play defensive roles such as acting as membrane-active antifungal, antibacterial, and antiviral agents (Tam et al., 2015; Dhama et al., 2018).

Many studies have been performed to evaluate pine needles (PN), and several compounds with antimicrobial activity have been detected (Ghaffari et al., 2019). The Korean red pine tree, *Pinus densiflora*, belongs to the family *Pinaceae* and is widely spread in East Asia (Korea, Japan, and China; Kim et al., 2020a). Various pine tree regions including needles, pollen, cones, and cortices are widely consumed as folk medicine, foods or dietary supplements for health promotion, antimicrobial, anti-inflammatory, and preservation effects (Hsu et al., 2006; Jeong et al., 2009). Pine bark protects collagen from the action of collagenase, while PNs exhibit anti-hypertensive effects and protect against oxidative DNA, protein, and lipid damage and oxidative stress-mediated apoptosis induced by hydroxyl radicals (Ku et al., 2007; Kim et al., 2010; Maimoona et al., 2011). Pine bark extracts are effective scavengers of free radicals and reactive oxygen species, can lower lipid levels in blood serum and may help to prevent disease and delay aging (Schafer et al., 2006; McGrath et al., 2015). PNs of form *P. densiflora* were used for folk medicine and for various disease prevention such as rheumatitis, hemorrhage, gastroenteric trouble, hypertension, and asthma (Kwak et al., 2006; Kim et al., 2020b). Recent scientific researches have shown that PNs form *P. densiflora* have antimicrobial, anti-viral, antioxidant, anti-mutagenic, anti-thrombosis, anti-asthmatic, and anti-inflammatory, and anti-cancer effects (Kwak et al., 2006; Park and Lee, 2011; Park et al., 2016; Ahn et al., 2018; Mostafa et al., 2018; Ha et al., 2020). However, the isolation and functional characterization of AMPs from PNs have been limited studies. Therefore, this study was conducted to identify and characterize AMPs from the needles of *P. densiflora* Sieb. et Zucc. We evaluated PN peptides to determine their antimicrobial ability against foodborne bacteria.

## Materials and Methods

### Pine Needle Collection and Extract Preparation

Fresh PNs of *P. densiflora* Sieb. et Zucc. were collected from Gok-Seong, Jeollanam-Do, Korea. The plant was initially determined based on its specific morphological observations, and the morphological and morphometric data were stored at the College of Natural Science and Public Health and Safety, Chosun University, Korea.

Pine needles of *P. densiflora* were rinsed with distilled water and then dried at 45°C for 5 h, and powdered using a mixer. The powder that passed through a 20-mesh sieve (850 μm) and was retained on a 40-mesh (450 μm) sieve, the mean particle size powder was stored in a sealed plastic bag at 25°C. PN powder (100 g) was added to 600 ml buffer (10 mM HEPES + 10 mM NaCl, pH 7.4) with continuous stirring at 25°C for 2 h. Thereafter, the extract was filtered and condensed under a vacuum in a rotary evaporator (N-1000VW, EYELA, Tokyo, Japan). The dried PNs extract was stored at 4°C until further analysis.

### Purification of Antimicrobial Proteins

**Figure 1:**
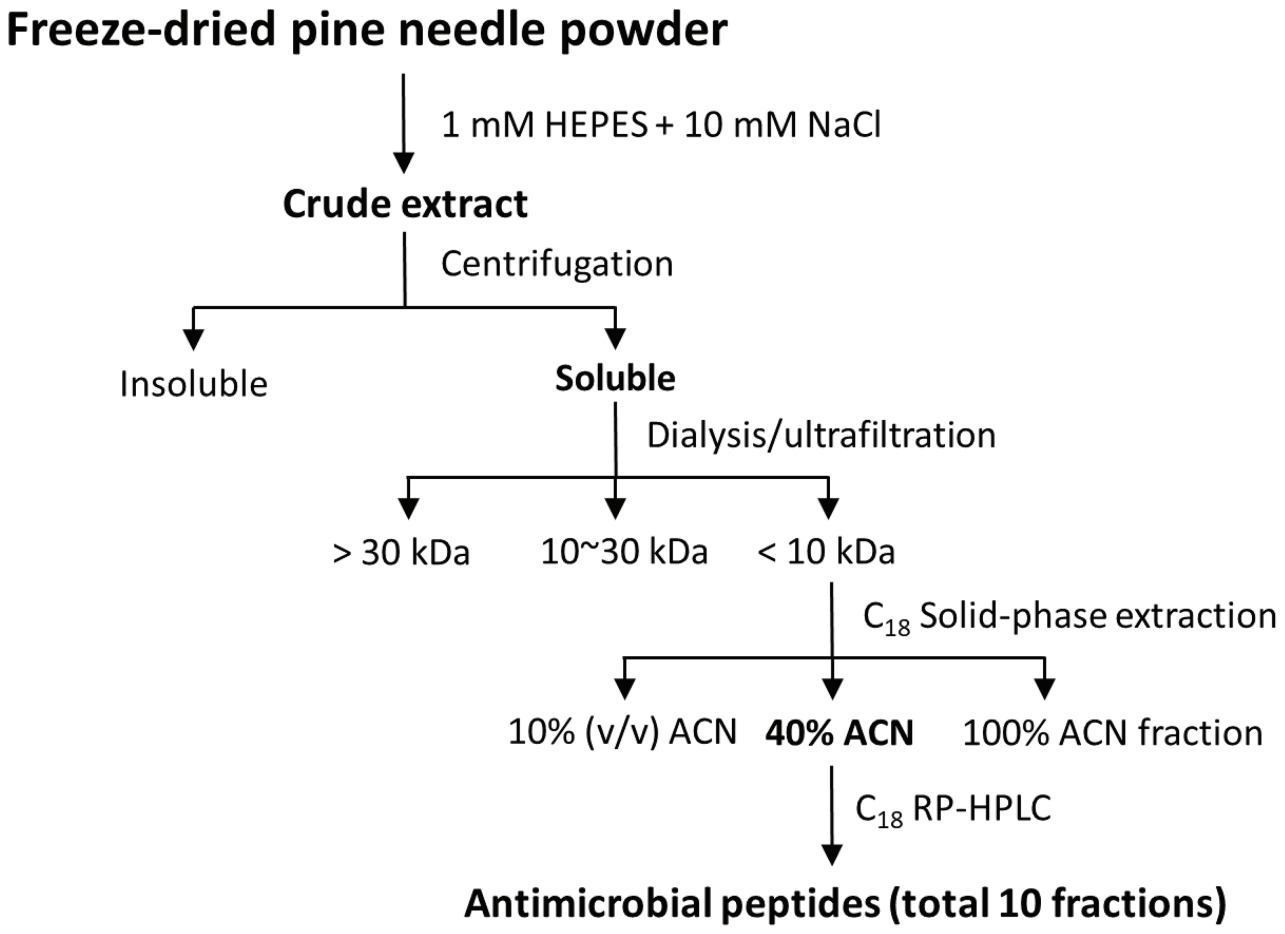
Separation strategy of AMPs from pine needles.

The dried extract sample was dissolved in distilled water and dialyzed using a molecular weight 1,000 membrane at 4°C overnight, and the dialyzed PN samples were purified using ultrafiltration (MW 30,000 and MW 1,000) and freeze-dried. The samples (MW < 10,000) were purified using an SPE 900 mg Lrg pore C_18_ column [Maxi-Clean™, Alltech Associates, Inc., Deerfield, IL, United States; 10% acetonitrile (ACN), 40% ACN, and 100% ACN]. The extract (40% ACN) was isolated using a reverse-phase C_18_ HPLC column [Jupiter 5u C_18_ 300A, 250 mm (length) × 4.6 mm (inner diameter), 300 Å pore size, 5 μm particle size] on an HPLC system (Shimadzu Corporation, Kyoto, Japan) that had been equilibrated using 0.1% (v/v) trifluoroacetic acid (TFA, Merck, Kenilworth, NJ, United States) in water with 5% ACN. PN peptide fractions were eluted using a linear gradient of solvents A and B were 0.1% (v/v) TFA in water and 0.1% (v/v) TFA in acetonitrile, respectively. Elution was carried out using a linear gradient of 40% solvent B for 10 min, 40–65% for 25 min, and 65–95% for 45 min at a flow rate of 1 ml/min. The eluates were monitored by measuring the absorbance signal at 215 nm (Figure 1). Individual fractions were pooled and then were freeze-dried at −20°C. To confirm partly as a single peptide, freeze-dried fractions were successively re-subjected to a second C_18_ HPLC at the peak of the overlapping point. Each fraction was collected and subsequently assayed for antimicrobial activities. The purified PN peptides were confirmed as a single molecule using tricine-sodium dodecyl sulfate-polyacrylamide gel electrophoresis (SDS-PAGE) and mass spectrometry.

### Tricine-SDS-PAGE

Tricine-SDS-PAGE is an efficient method of separating low-molecular-mass peptides (16.5% polyacrylamide gel for peptide <10 kDa; Schagger and von Jagow, 1987). The purified PN peptides were resolved in a 16.5% tricine-SDS PAGE, followed by visualization with Coomassie Brilliant Blue G-250 staining.

### Mass Spectrometry

Matrix-assisted laser desorption ionization-time of flight mass spectrometry (MALDI-TOF-MS) was carried out using an Axima-CFR MALDI-TOF mass spectrometer (Kratos Analytical, Manchester, United Kingdom) as described by Pouvreau et al. (2001). The protein concentration of the purified PN peptide was determined by the Bradford assay using bovine serum albumin (BSA) as the calibration standard (Bradford, 1976).

### PN Peptide Identification

The *N*-terminal amino acid (a.a.) sequences of the purified PN peptide was determined with an automated Edman degradation method using a pulse liquid automatic sequencer (Procise Model 491 HT protein sequencer; Applied Biosystems, Foster City, CA, United States) at the Korean Basic Science Institute (Seoul, Korea). The PN peptide sequences were compared to those in the Antimicrobial Peptide Database (APD), using the “APD3: Antimicrobial Peptide Calculator and Predictor” tool (Wang et al., 2016).

### Antimicrobial Assay

For antimicrobial assays, *Escherichia coli* ATCC 25922, *Pseudomonas aeruginosa* ATCC 15692, *Staphylococcus aureus* ATCC 25923, and *Staphylococcus epidermidis* ATCC 12228 were obtained from the American Type Culture Collection (Manassas, VA, United States). *Salmonella typhimurium* KCTC 1926 and *Listeria monocytogenes* KCTC 3710 were obtained from the Korean Collection for Type Cultures (Jeollabuk-do, Korea).

Minimum inhibitory concentrations (MICs) of purified PN peptides were assayed according to the Clinical and Laboratory Standards Institute recommendations (Wiegand et al., 2008). Briefly, bacterial suspension (5 × 10^5^ CFU/ml), obtained by diluting an exponential growth phase culture, was then added into the 96-well plates containing 2-fold serial dilutions of each fraction, the plates were incubated for 18 h at 37°C. MICs of PN peptides were measured in optical density at 600 nm using Versamax™ ELISA Microplate Reader (Molecular Devices, Sunnyvale, CA, United States). PBS, culture media, and melittin were used as growth and growth inhibition controls. The MIC was defined as the lowest concentration of PN peptide that was able to inhibit microbial growth.

### Hemolytic Activity

Hemolysis was determined using the mouse red blood cells (mRBCs). Fresh mRBCs were collected in PBS and washed three times with PBS (final mRBC concentration, 8% v/v). PN peptides were assessed at 200 μM final concentration and incubated for 60 min at 37°C. Hemolytic activity was monitored at an absorbance (A) of 414 nm and calculated as a positive control [0.1% Triton X-100 (A_triton_)] and negative control [PBS (A_blank_)]. The resulting values of cells that underwent hemolysis were calculated according to equation (Park et al., 2008):

(1) $$\mathrm{Hemolysis}=[\left(A_{\mathrm{sample}}-A_{\mathrm{blank}}\right)/\left(A_{\mathrm{triton}}-A_{\mathrm{blank}}\right)]\times 100\%$$

### Cytotoxicity

The cytotoxic effect of PN peptides was assessed against HaCaT (human keratinocytes) and RAW264.7 (mouse macrophages) cells cultured in 96-well plates at a density of 2 × 10^4^ cells/well in Dulbecco’s modified Eagle medium (DMEM) containing 10% fetal bovine serum. MTT [3-(4,5-dimethylthiazol-2-yl)-2,5-diphenyltetrazolium bromide] assays were used to evaluate cytotoxicity. PN peptides (0–200 μg/ml) were added with the cells for 24 h at 37°C. Subsequently, MTT (0.5 mg/ml) was added to each well and incubated for 4 h at 37°C After incubation, formazan crystals produced was dissolved in dimethyl sulfoxide, and the absorbance at 570 nm was measured and cytotoxicity was determined as a percentage of 100% cytotoxic control (0.1% Triton X-100; Park et al., 2008). Melittin was used as the control (reference) peptide.

### Computational Analyses

3-D structural projections of PN5 were created online using the Mobyle@RPBS bioinformatics portal.^1^ The helical wheel projection was obtained using the online tool HeliQuest^2^ (Gautier et al., 2008).

### Peptide Synthesis

PN5 peptides were synthesized using the fluorenylmethoxycarbonyl (Fmoc) solid-phase peptide synthesis on a solid support of rink amide 4-methylbenzhydrylamine resin (Merck KGaA, Darmstadt, Germany) with Liberty microwave peptide synthesizer (CEM Co., Matthews, NC, United States; Kang et al., 2018). For coupling reactions, 0.1 M *N*-hydroxybenzotriazole and 0.45 M 2-(1H-benzotriazole-1-yl)-1,1,3,3-tetramethyluronium hexafluorophosphate in dimethylformamide (DMF) and 2 M *N*,*N*-diisopropylethylamine in *N*-methylpyrrolidone were used as coupling reaction solutions, and a 10-fold Fmoc-protected a.a. (Novabiochem, Läufelfingen, Switzerland) was added during all coupling reaction cycles. Fmoc from the Fmoc-protected synthetic peptide was cleaved with 20% (v/v) piperidine in DMF. After cleavage from resin, the crude PN peptides were purified using RP-HPLC on a Jupiter C_18_ column (250 mm × 21.2 mm, 15 μm, 300 Å) with a 0–60% ACN gradient in water containing 0.05% TFA. The purity of the peptides (>95%) was then determined by an analytical RP-HPLC using a Jupiter proteo C_18_ column (250 mm × 4.6 mm, 90 Å, 4 μm). The molecular weights of the synthetic peptides were detected by MALDI-TOF MS (MALDI II; Kratos Analytical, Inc., Chestnut Ridge, NY, United States).

### Statistical Analysis

All experimental data represent mean ± standard deviation (SD). All the figures were obtained from several independent experiments and showed similar results. Differences among groups were analyzed using one-way ANOVA followed by Tukey’s multiple-comparison test.

## Results

### Purification of AMPs From Pine Needles

**Figure 2:**
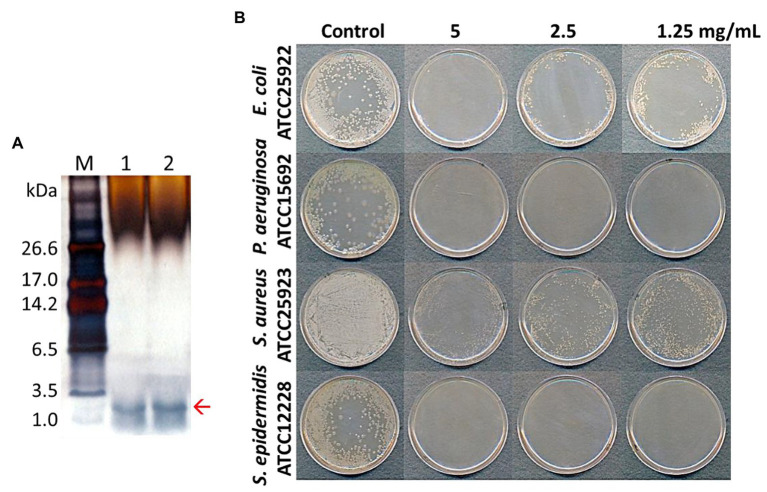
Tricine-SDS-PAGE analysis of total protein extracted from pine needles and evaluation of their antibacterial activity against bacterial strains [lane M; molecular weight size marker, Lanes 1 and 2: total extracted proteins from pine needles (5 and 10 μg, respectively)] **(A)**; Antimicrobial activity of extracted protein fraction against *Escherichia coli*, *Pseudomonas aeruginosa*, *S. aureus*, and *Staphylococcus epidermidis* **(B)**. The red arrow indicates the expected PN peptides.

AMP fractions were purified from PNs in five steps: extraction, dialysis, ultrafiltration, purification using SPE 900 mg Lrg pore C_18_ column, and C_18_ reverse-phase HPLC (Figure 1). By varying the concentration of the sample separated using Tricine gel electrophoresis, small proteins with sizes of 1–3.5 kDa were obtained (Figure 2A). The antimicrobial activity of the dialyzed samples was tested against pathogenic bacteria (*E. coli*, *S. aureus*, *P. aeruginosa*, *S. aureus*, and *S. epidermidis*) and bacterial strains that cause food poisoning (*S. typhimurium* and *L. monocytogenes*). Results displayed that the dialyzed samples exhibited potent antimicrobial activity toward all tested bacteria at low concentrations (>5 mg/ml; Figure 2B; Supplementary Figure S3). The dialyzed samples were isolated using ultrafiltration (MW 30,000 and 10,000). According to the results shown in Supplementary Figure S1, all ultrafiltered fractions (>30, 10–30, and <10 kDa) inhibited *E. coli* and *S. aureus*. Among these fractions, the antimicrobial activity of the sample ≤10 kDa in size was the highest. In addition, this fraction was most effective in inhibiting *S. aureus* (2.5 mg/ml) compared to *E. coli* (5 mg/ml) and showed the same results as the dialyzed samples. After confirming the antimicrobial activity against *L. monocytogenes* and *S. typhimurium* of the ultrafiltered fractions ≤10 kDa, we found that these samples retained the same activity as the dialyzed samples (Supplementary Figure S3). As shown in Table 1, the MIC value increased considerably after the first purification step using ultrafiltration (1.5-fold compared to the soluble extract). The total protein content (512 mg) was lower than that of the initial soluble extract of PNs (1,250 mg).

**Table 1:** Purification scheme for AMPs from water-soluble extract of pine needles.

Purification step*^a^*	Volume (ml)	Protein (μg/ml)*^b^*	Total protein (mg)*^c^*	MIC (mg/ml)*^d^*	Increased in MIC (fold)	Recovery per step (%)
Soluble extract	500	1,500	750	7.5	-	100
Ultrafiltration	380	920.6	349.8	5	1.5	46.7
C_18_ solid-phase extraction	274	350.7	96.1	3	2.5	12.9
(PN-#5)						
First C_18_ RP-HPLC	10.5	282.8	3.0	0.6	12.5	0.4
Second C_18_ RP-HPLC	6.5	273.9	1.8	0.032	234.4	0.24
(PN-#7)						
First C_18_ RP-HPLC	10.3	274.6	2.83	1.0	7.5	0.38
Second C_18_ RP-HPLC	6.5	196.8	1.28	0.064	117.2	0.17
(PN-#8)						
First C_18_ RP-HPLC	10.4	176.9	1.84	0.9	8.3	0.25
Second C_18_ RP-HPLC	6.3	154.3	0.97	0.064	117.2	0.13
(PN-#10)						
First C_18_ RP-HPLC	10.1	305.1	3.08	0.4	18.8	0.41
Second C_18_ RP-HPLC	6.0	287.4	1.72	0.008	937.5	0.23

a Small-scale separation and purification experiments were performed several times using 1-ml column and then expanded to a larger volume column (20 ml).

b Total protein.

c Protein concentration was measured using the Bradford method using BSA as a standard.

d MIC of *Staphylococcus aureus* ATCC25923.

**Figure 3:**
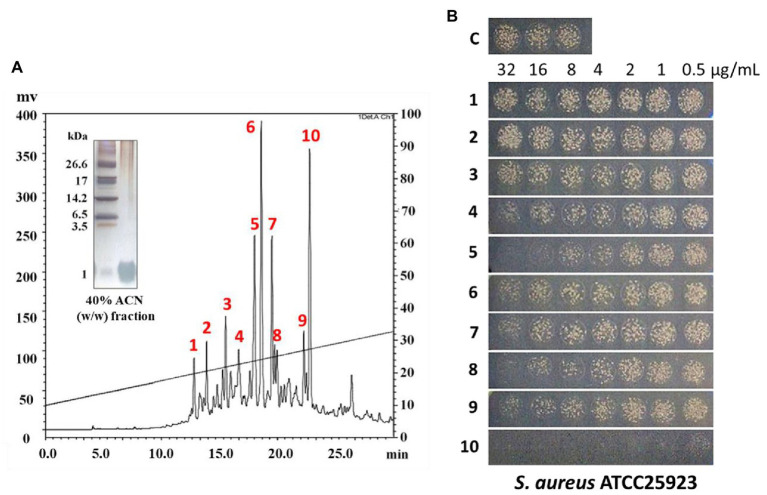
RP-HPLC analysis of peptides from 40% (w/w) ACN fractions of pine needles and estimation of their antimicrobial activity against *Staphylococcus aureus*. RP-HPLC chromatograms (absorbance was monitored at 215 nm) **(A)**; Peak fractions from 40% (w/w) ACN fractions of pine needles exhibiting antimicrobial activity against *Staphylococcus aureus* **(B)**.

In this study, the small (≤10 kDa) sample was treated as follows. The active ultrafiltered fractions of the sample ≤10 kDa were applied to an SPE 900 mg Lrg pore C_18_ column. The fractions from solid-phase extraction were collected using the following stepwise gradient: 10, 40, and 100% ACN (w/w). The 40% ACN (w/w) fraction was found to have antimicrobial activity (Supplementary Figures S2, S3C). C_18_ solid-phase extraction resulted in a 1.6-fold increased MIC value. The total protein (34.8 mg) decreased and the recovery was 2.8% of that for the soluble extract (Table 1). The active 40%-ACN (w/w) fractions were subjected to C_18_ RP-HPLC twice. Ten main peaks with retention times of 12–25 min were detected, which were eluted over a 40–65% gradient (Figure 3A). These peak fractions were checked for antimicrobial activity and the active PN peptide peaks were collected, pooled (Figure 3B), and confirmed using Tricine-SDS-PAGE, which resulted in one peptides band with molecular weights of 1–3.5 kDa (Figure 3A). The first elution yield of total protein obtained after the RP-HPLC step was drastically reduced (0.25–0.41%), although the MIC value against *S. aureus* was higher than that of the soluble extract (7.5–18.8-fold). The second RP-HPLC step increased the MIC value of the antibacterial peptides (PN-#5, PN-#7, PN-#8, and PN-#10) 117.2–937.5-fold compared to the initial soluble extract, with a recovery of 0.13–0.24% compared to the soluble extract (Table 1).

**Figure 4:**
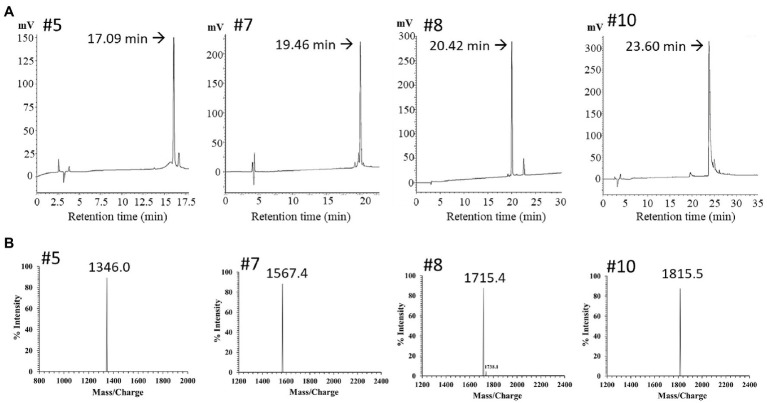
RP-HPLC and mass spectrum of purified peak peptides (PN-#5, PN-#7, PN-#8, and PN-#10). Chromatogram of AMPs in the 40% ACN fraction obtained using HPLC **(A)**; Mass spectra of purified peak peptides using MALDI-TOF/MS **(B)**.

The PN peptide fractions with potent antimicrobial activity (PN-#5, PN-#7, PN-#8, and PN-#10) were analyzed using RP-HPLC (Figure 4A) and MALDI TOF-MS (Figure 4B). MALDI TOF-MS analysis showed that PN-#5, PN-#7, PN-#8, and PN-#10 exhibited m/z peaks at 1346.0, 1567.4, 1715.4, and 1815.5, respectively (Figure 4B).

### Antimicrobial Assay of PN Peptides

The bactericidal activities of PN peptides (PN-#1–10) were tested against *S. aureus* and *E. coli* (Figure 3B). None of the peptide fractions, except PN-#10 (data not shown), showed antimicrobial activity against *E. coli* up to a concentration of 32 μg/ml. In contrast, PN-#5, PN-#7, PN-#8, and PN-#10 showed particularly potent activities against *S. aureus* (Figure 3B). PN-#1, PN-#2, and PN-#3 exhibited no activity against *S. aureus* and *E. coli*. We determined the MIC values of PN-#5, PN-#7, PN-#8, and PN-#10 for *S. aureus*, *E. coli*, *L. monocytogenes*, and *S. typhimurium* (Table 2). The MIC values of the PN peptides ranged from 8 to 128 μg/ml. Of all PN peptides from PNs, PN-#10 showed the best activity against all tested bacteria, with MIC values of 8, 16, 32, and 32 μg/ml for *S. aureus*, *L. monocytogenes*, *E. coli*, and *S. typhimurium*, respectively. As a result, all PN peptides were more susceptible to Gram-positive bacteria (*S. aureus* and *L. monocytogenes*) than Gram-negative bacteria (Table 2).

**Table 2:** Antimicrobial activities of purified PN peptides.

	MIC (μg/ml)*^a^*			
	PN-#5	PN-#7	PN-#8	PN-#10
Gram-positive bacteria				
*Staphylococcus aureus* ATCC25923	32	64	64	8
*Listeria monocytogenes* KCTC3710	32	64	64	16
Gram-negative bacteria				
*Escherichia coli* ATCC25922	64	128	128	32
*Salmonella typhimurium* KCTC1926	64	128	128	32

a MIC refers to the minimal concentration of peptide to inhibit microbial growth.

### Hemolytic Activity and Cytotoxicity of PN Peptides

**Figure 5:**
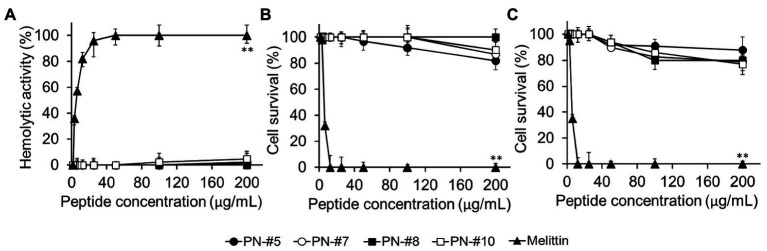
Hemolysis in mRBCs and cytotoxicity against RAW264.7 murine macrophages and HaCaT human keratinocyte cells. Hemolytic activity against mRBCs (*n* = 3 per condition) **(A)**. Cytotoxic activity against RAW264.7 (*n* = 4 per condition) **(B)**; and HaCaT (*n* = 3 per condition) **(C)** cells. Statistical analysis was performed by one-way ANOVA. **p < 0.01 vs. melittin.

The toxicity of the AMPs against eukaryotic cells was evaluated based on their lytic ability at tested concentrations (0–200 μg/ml; Figure 5A). The percentage of hemolysis was determined by measuring the amount of mouse hemoglobin released after incubation with PN-#5, PN-#7, PN-#8, PN-#10, or melittin (the reference AMP). The PN peptides did not cause hemolysis even at a concentration of 200 μg/ml. In contrast, melittin induced hemolysis even at the lowest concentration (3 μg/ml).

The cytotoxicity of the peptides against RAW264.7 murine macrophages and HaCaT keratinocytes is shown in Figures 5B,C, respectively. The PN peptides at a concentration of 200 μg/ml caused less than 20% cytotoxicity in the RAW264.7 and HaCaT cell lines. These results suggested that the PN peptides are not toxic in the MIC range. In contrast, melittin, the reference AMP, caused 100% lysis at a concentration of 12.5 μg/ml (Figures 5B,C).

### PN5 Identification

To identify the peptides and their a.a. sequence, PN-#5 was analyzed *N*-terminal a.a. sequence analysis with an Edman degradation. The a.a. sequence of the purified PN5 peptide was determined to be FKFLARTGKFL. BLASTp database search of the PN5 peptide was carried out to identify regions of similarity between NCBI sequence databases. The results revealed that the a.a. sequence FKFLARTGKFL has only two a.a. differences from FKYLQRTGKFL of transposase (NCBI seq. Id WP_065256059) from *Moraxella lacunata*.

For potential AMP prediction and their similarities to database-defined AMPs, the “APD3: Antimicrobial Peptide Calculator and Predictor” tool of the APD was used to identify PN peptides (Wang et al., 2016). The PN5 sequence showed 50% similarity with Temporin-HB2 from the Hubei gold-striped pond frog *Pelophylax hubeiensis* found in China (APD ID: AP02838, FLPFLAGLFGKIF), and Temporin-1Ce from the bronze frog *Rana clamitans* (APD ID: AP00108, FLPFLATLLSKVL). However, the sequence of PN5 did not identify any other plant protein or peptide, revealing that PN5 is a novel plant AMP. Figure 4B (#5) displays the singly charged potassium adducts [M+K]^+^ of PN5 with an *m/z* value of 1,347, equivalent to 1,328 Da, was determined using MALDI-TOF/MS. These results and the theoretical molecular weight of PN5 agree well with the determined a.a. sequence.

### PN5’s Secondary Structure and in silico Analysis

**Figure 6:**
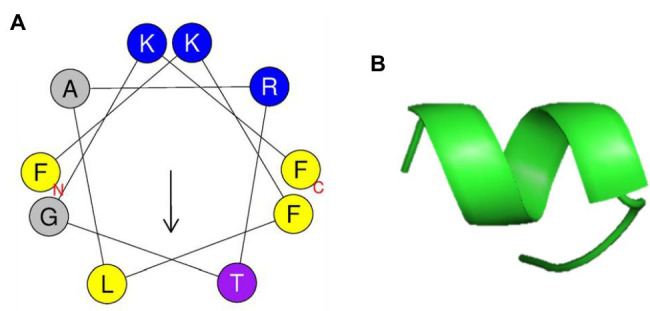
Helical wheel projection and three-dimensional structure projection of PN5 peptide. Helical wheel projection of PN5 was generated using HeliQuest (http://heliquest.ipmc.cnrs.fr/cgi-bin/ComputParams.py) **(A)**. Simulations of the three-dimensional structures of peptides generated using PyMOL **(B)**.

PN5 has an α-helical amphipathic structure, as suggested by the helical wheel projection in Figure 6A. In addition, the PN5 peptide carried a net charge (3) and was hydrophobic (H, 0.465). The ExPASy tool (SIB Bioinformatics Resource Portal) and APD3 (Antimicrobial Peptide Calculator and Predictor) were used to obtain the physicochemical properties of PN5 such as theoretical isoelectric point (pI, 11.17), hydrophobic moment (μH, 0.471), protein-binding potential (Boman index, 0.64 kcal/mol), and grand average of hydropathicity (GRAVY, 0.400). The PN5 instability index was estimated to be −20.01, indicating a stable peptide under both *in vitro* and *in vivo* conditions. These results with *in silico* prediction were consistent with the results obtained using PyMOL, which predicted that PN5 assumes an α-helical conformation (Figure 6B).

### PN5 Peptide Synthesis, Antimicrobial Activity, and Toxicity

In general, amidated peptides have a greater tendency to generate an α-helical structure than the non-amidated peptides. Chemically synthesized peptides with *C*-terminal amidation showed strong inhibitory activity against bacteria and low hemolytic activity (Strandberg et al., 2007; Shahmiri et al., 2015). Thus, we synthesized PN5 peptide (FKFLARTGKFL) and PN5 with an amidated *C*-terminus (FKFLARTGKFL-NH_2_), based on the *N*-terminal sequence results.

The antimicrobial activities of the synthetic PN5 and PN5-NH_2_ peptide were evaluated against two Gram-negative (*E. coli* and *S. typhimurium*) and two Gram-positive (*S. aureus* and *L. monocytogenes*) bacteria. The synthetic PN5 peptide showed the same antimicrobial activity as that did the purified PN5 peptide (PN-#5; Table 3). PN5-NH_2_ peptide showed better antimicrobial activity than PN-#5, against *S. aureus*, *L. monocytogenes*, *E. coli*, and *S. typhimurium* with MICs of 16, 32, 64, and 32 μg/ml, respectively (Table 3).

**Table 3:** Antimicrobial activities of synthetic PN5 against foodborne bacteria.

	MIC (μg/ml)^a^		
	PN5	PN5-NH_2_	Melittin
Gram-positive bacteria			
*Staphylococcus aureus* ATCC25923	32	16	4
*Listeria monocytogenes* KCTC3710	32	32	4
Gram-negative bacteria			
*Escherichia coli* ATCC25922	64	64	2
*Salmonella typhimurium* KCTC1926	64	32	2

a MIC refers to the minimal concentration of peptide to inhibit microbial growth.

After analyzing the synthetic PN5 activity, to confirm the increased toxicity of PN5-NH_2_, hemolysis and cytotoxicity were confirmed under the same conditions as those used for the purified PN-#5 peptide (Supplementary Figure S4). The synthetic PN5 peptide showed only approximately 5% hemolysis activity at a high concentration of 200 μg/ml, which is similar to that of the PN-#5 peptide (Supplementary Figure S4A). The PN5 peptide caused only 19% cytotoxicity up to a concentration of 200μg/ml in the HaCaT cell line (Supplementary Figure S4B). These results show that the synthetic PN5 peptide is not toxic in the MIC range, which is consistent with the toxicity of the PN-#5 peptide. Thus, the PN5 peptide appears to be a good candidate as an AMP.

## Discussion

Recent studies on PN extract have been reported as food and health supplements, as well as food preservatives (Mahajan et al., 2016; Wu et al., 2017). Contaminated food by the pathogenic microorganisms is a major common form of food degradation, and is often responsible for the occurrence of food-borne illnesses such as infection, food poisoning, toxic shock syndrome, and sepsis (Bintsis, 2017; Chen and Alali, 2018; Riley, 2020). Increasing the use of chemical preservatives can effectively prevent the survival and proliferation of most food-borne bacteria; however, drawbacks related with the safety of chemical preservatives are on the rise (Halla et al., 2018; Silva et al., 2018). Recently, as the demand for foods with health benefits has increased, the amount of chemical preservatives used in foods has decreased; thus, there is a need for natural preservatives. As bacterial resistance to antibiotics is increasing, many studies have been performed to develop effective and nontoxic antimicrobial substances that do not induce antimicrobial resistance. Many studies are being conducted to develop new natural antibacterial substances in various plant extracts for safe food preservation and to investigate whether they have antibacterial activity in various microorganisms including food-borne bacteria (Hintz et al., 2015; Mostafa et al., 2018). Recent studies reported new AMPs (peptides consisting of 10–14 a.a. residues) isolated from the Mediterranean medical plant *Charybdis*, with similar antimicrobial activity (25–45 μ/ml in *S. aureus* and *P. aeruginosa*) as that of PN peptides (Cunsolo et al., 2020). In this study, the PN peptide showed potent antimicrobial activity against some representative food poisoning pathogens, particularly *S. aureus* (Figure 3B; Table 2). PN peptides had better antimicrobial activity against Gram-positive bacteria than Gram-negative bacteria, and showed the highest ability to kill *S. aureus*. This may be related to differences in bacterial cell structure between Gram-positive and Gram-negative bacteria. The cell wall of Gram-negative bacteria is thinner but more complex than that of Gram-positive bacteria. It has been suggested that differences in antimicrobial activity toward some antimicrobial agents between Gram-positive and Gram-negative bacteria are due to the low permeability of Gram-negative bacteria to antibacterial compounds through the outer cell wall. The outer cell wall with low permeability of Gram-negative bacteria can interfere with the accumulation in the cytoplasmic membrane, thereby reducing the killing efficiency of the antimicrobial compound (Zgurskaya et al., 2015; Breijyeh et al., 2020). Besides transporting small molecules such as nutrients, the membranes of Gram-negative bacteria are resistant to chemical entry (Wu et al., 2016; Tiz et al., 2018). Therefore, developing alternative substances is essential for eradicating these bacteria using natural AMPs.

PN peptides isolated from PNs from *P. densiflora* Sieb. et Zucc. exhibited strong antimicrobial activity against foodborne bacteria. Moreover, the AMPs PN-#5, PN-#7, PN-#8, and PN-#10 exhibited no cytotoxicity at the antimicrobial concentrations. Given these results, PN5 might become an effective drug candidate for developing antimicrobial agents in the food and pharmaceutical industries. In addition, PN5 peptide can be used as a natural food additive. Future studies can be directed at a.a. substitution on the primary structure, to obtain more active AMPs.

## Data Availability Statement

The datasets presented in this study can be found in online repositories. The names of the repository/repositories and accession number(s) can be found in the article/Supplementary Material.

## Author Contributions

JL: study design, experiment, and data analysis. HKK: study design, experiment, data analysis, and writing – original draft. HC: methodology, visualization, and resources. YP: investigation, resources, and writing – review and editing. All authors contributed to the article and approved the submitted version.

### Conflict of Interest

The authors declare that the research was conducted in the absence of any commercial or financial relationships that could be construed as a potential conflict of interest.

## Footnotes

^1^http://mobyle.rpbs.univ-paris-diderot.fr/cgi-bin/portal.py#welcome

^2^http://heliquest.ipmc.cnrs.fr
