# Supplementary material for: A Novel Antimicrobial Peptides From Pine Needles of Pinus densiflora Sieb. et Zucc. Against Foodborne Bacteria
Source: Front Microbiol. 2021 May 20;12:662462. doi: 10.3389/fmicb.2021.662462 (PMC8172577; doi:10.3389/fmicb.2021.662462)
Supplement: Supplementary file 1 [file Data_Sheet_1.PDF]

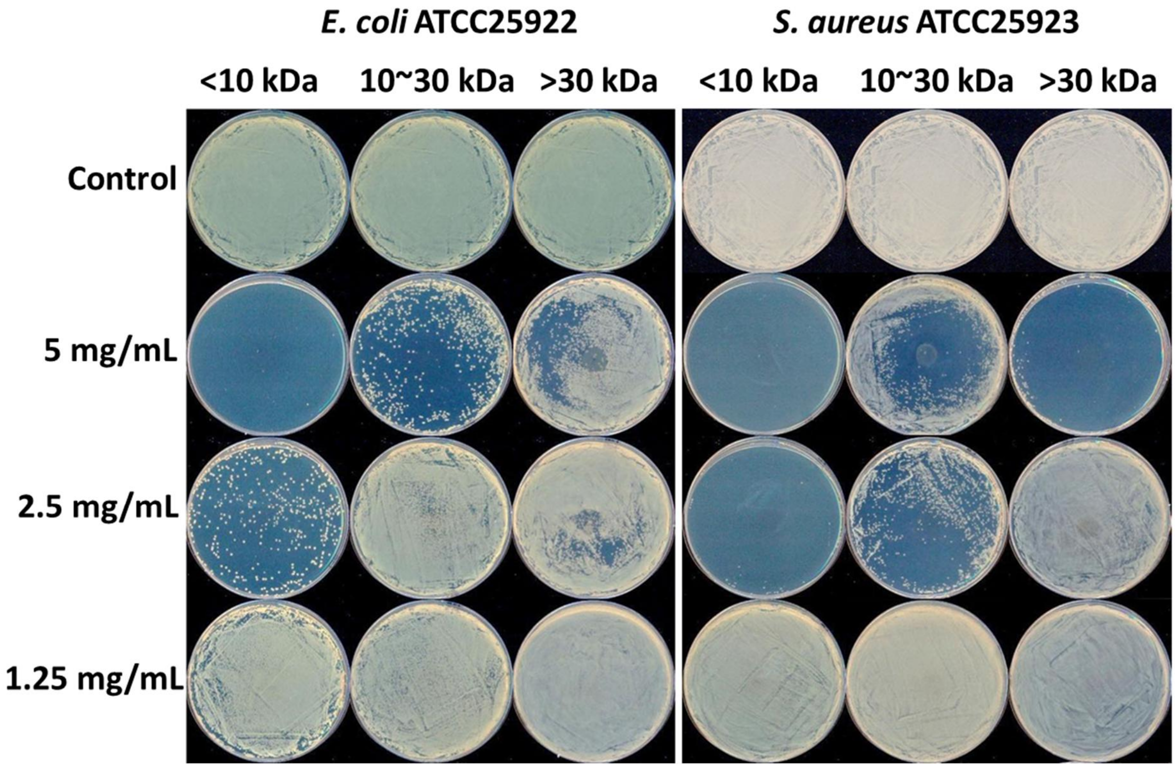

FIGURE S1 Antibacterial activity of ultrafiltrated fractions. The ultrafiltrated fractions (< 10kDa, 10~30 kDa and >30 kDa) exhibited antibacterial activity against *S. aureus* and *E. coli*.

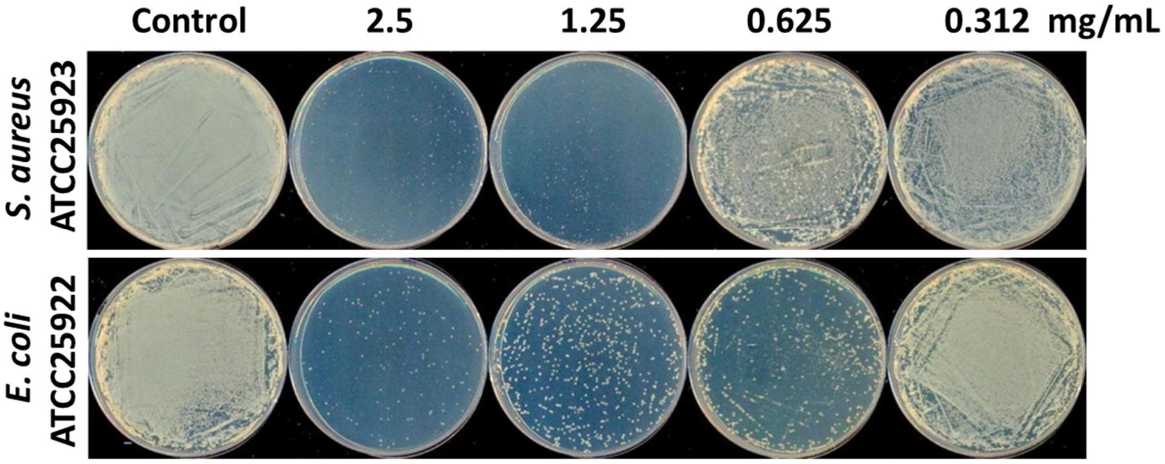

FIGURE S2 Antibacterial activity of purified protein by C_{18} Solid-phase extraction. The ACN 40 % (w/w) fraction exhibited antibacterial activity against *S. aureus* and *E. coli*.

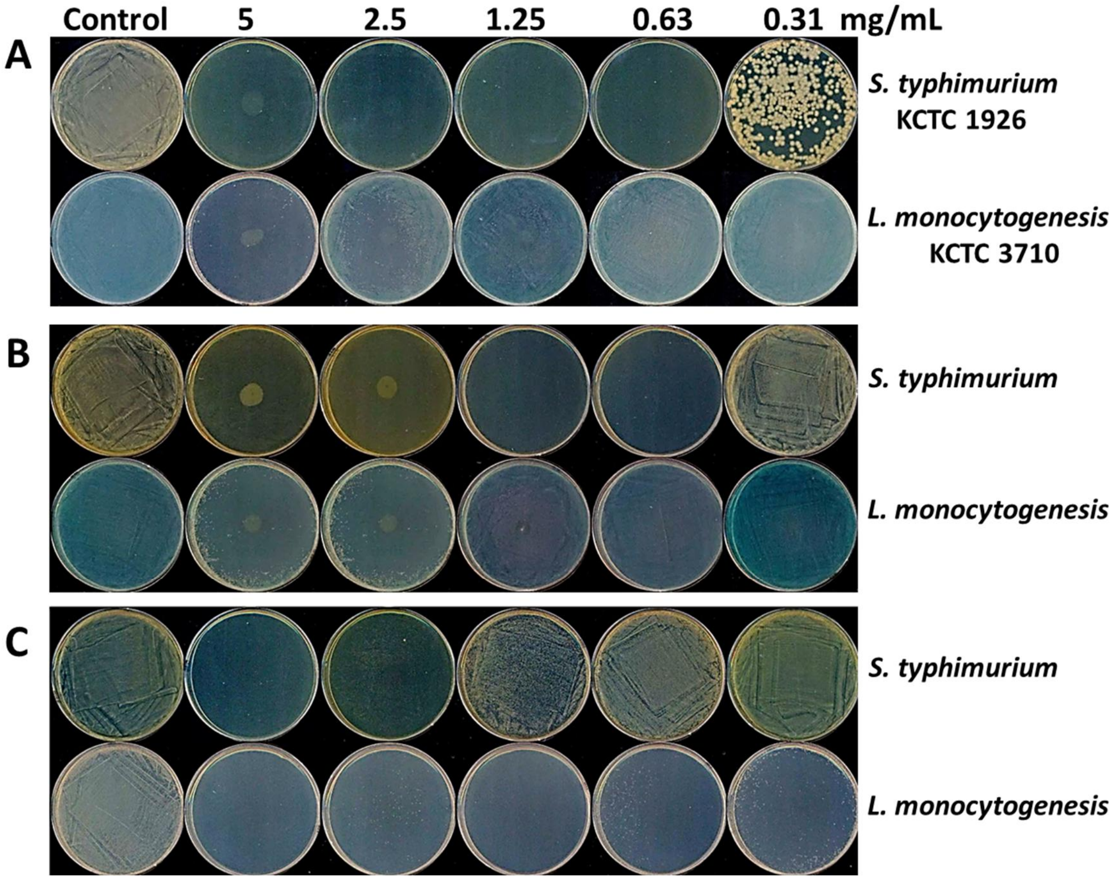

FIGURE S3 Antibacterial activity of each step fractions from pine needles against foodborne bacteria (*S. typhimurium* and *L. monocytogenes*). Total extracts (**A**); Utrafiltrated extracts (< 10kDa) (**B**); ACN 40 % (w/w) (**C**) fractions from pine needles.

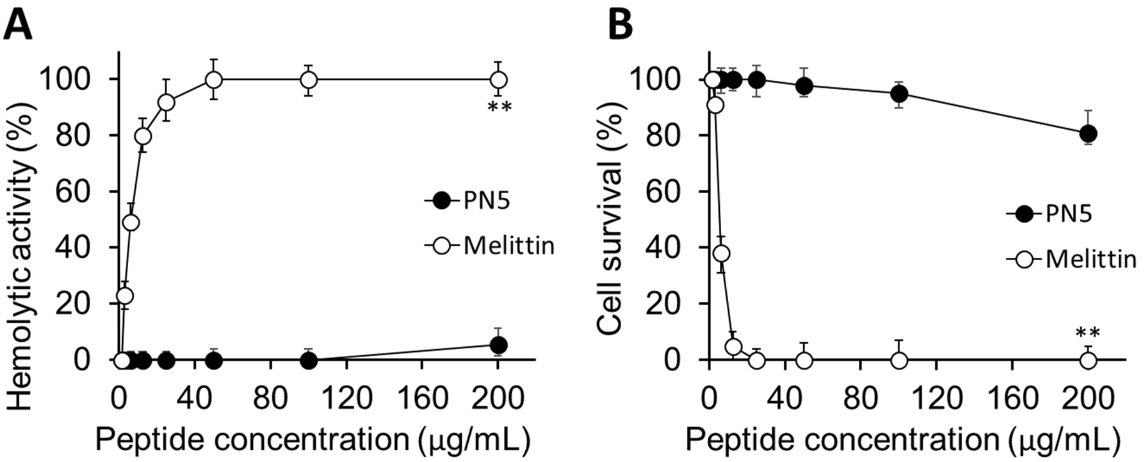

FIGURE S4 Hemolysis in mRBCs and cytotoxicity against HaCaT human keratinocyte cells of the synthetic PN5 peptide. Dose-response curve of hemolytic activity against mRBCs (n=3 per condition) (**A**). Dose-response curves for cytotoxic activity against HaCaT (n=3 per condition) cells (**B**). Symbols represent the means ± SD from triplicate determinations. Data were analyzed using one-way ANOVA. ***P* < 0.01 vs. melittin.
